# Comment to “ Until when will we grant specialist titles to doctors without medical residency?”

**DOI:** 10.1590/0100-6991e-20243827-en

**Published:** 2024-11-26

**Authors:** PAULO ROBERTO CORSI

**Affiliations:** 1 - Faculdade de Ciências Médicas da Santa Casa de São Paulo, Santa Casa de São Paulo - São Paulo - SP - Brasil

With due respect to the opinion of Dr. Francisco Arsego^1^, I believe that everything can be relativized, since the perceptions and interpretations of the facts vary according to the circumstances and the point of view of those who observe them. Although I appreciate his argument, it is important to consider that the context, nuances, and particularities of each situation can lead us to different conclusions, without a view necessarily invalidating the other.

The question raised about “how long will we allow a newly graduated doctor to work without any kind of supervision?” is extremely relevant and goes to the heart of a chronic problem. Almost all discussions about medical training and certification end in the same complex complicating factor: the criminal way in which medical schools have been treated in recent decades in Brazil. The large and unjustified increase in the number of schools, due to economic and political interests, together with the growth of spots in existing universities, often without the corresponding expansion of the infrastructure necessary to guarantee quality education, throw newly ill-trained professionals into the labor markets. Many institutions also face some serious shortcomings, such as a lack of qualified professors, inadequate laboratories, and the absence of teaching hospitals. Consequently, graduates of these institutions are often not able to act with the competence required by the profession.

Medical Residencies (MR) have not kept up with this growth and currently more than half of college graduates cannot find specialization spots. Obviously, indiscriminately opening unqualified MR vacancies will not adequately solve the problem.

MR is defined by Law 6.932 (1981) as a modality of postgraduate education aimed at physicians in the form of a specialization course, characterized by in-service training in health institutions, university or not, under the guidance of medical professionals with high ethical and professional qualifications. It is also known as an education system with a thousand and one defects and a single quality: it is, by far, the best way to train a specialist in medicine. Upon completing the residency program, the doctor automatically acquires the Title of Specialist.

However, the fact that most programs fail no one raises serious concerns about the real competence of trained professionals, and the absence of a robust evaluation mechanism can compromise the quality of specialized medical care. Residency by itself should not be a guarantee of title, because, in many cases, it does not offer the quality or requirement necessary to train a truly qualified specialist.

The Brazilian College of Surgeons, in partnership with the Brazilian Medical Association, has been providing the Title of Specialist in General Surgery for more than 30 years, through serious and rigorous tests, with a high failure rate. Prerequisites are required for registration and there are two more phases: written and oral tests. A few years ago, a practical phase was also included, with clinical cases and technical skills stations. 

According to Decree 8516 (2015), there is a legal equivalence between the Title of Specialist of the MR recognized by the Ministry of Education (MEC) and of the Medical Societies, although civil society gives greater importance to the title of the societies. For this reason, even those with the MEC title take the Society/AMB exam.

Perhaps the most important questions are:

- How long will we allow the number and operation of inadequate medical schools to increase? and
- Until when will we provide the title of specialist in a notarial manner, without any verification?
